# Enhancing Surgical Vision: Augmented Reality in Otolaryngology—Head and Neck Surgery

**DOI:** 10.1089/jmxr.2024.0010

**Published:** 2024-07-09

**Authors:** Marina Aweeda, Feyisayo Adegboye, Shiayin F. Yang, Michael C. Topf

**Affiliations:** 1 Department of Otolaryngology—Head and Neck Surgery, Vanderbilt University Medical Center, Nashville, Tennessee, USA.; 2 School of Engineering, Vanderbilt University, Nashville, Tennessee, USA.

**Keywords:** otolaryngology, augmented reality, head and neck surgery, AR, surgical technology

## Abstract

Augmented reality (AR) technology has become widely established in otolaryngology—head and neck surgery. Over the past 20 years, numerous AR systems have been investigated and validated across the subspecialties, both in cadaveric and in live surgical studies. AR displays projected through head-mounted devices, microscopes, and endoscopes, most commonly, have demonstrated utility in preoperative planning, intraoperative guidance, and improvement of surgical decision-making. Specifically, they have demonstrated feasibility in guiding tumor margin resections, identifying critical structures intraoperatively, and displaying patient-specific virtual models derived from preoperative imaging, with millimetric accuracy. This review summarizes both established and emerging AR technologies, detailing how their systems work, what features they offer, and their clinical impact across otolaryngology subspecialties. As AR technology continues to advance, its integration holds promise for enhancing surgical precision, simulation training, and ultimately, improving patient outcomes.

## Introduction

In the ever-evolving landscape of medical advancement, augmented reality (AR) technologies have emerged as a new player. Although originally used for immersive experiences in entertainment,^1^ AR has made significant strides in medicine,^2^ particularly in the field of otolaryngology—head and neck surgery. AR exists on the extended reality (XR) spectrum, which includes virtual reality (VR), AR, and mixed reality (MR). Unlike VR, which immerses the user in a completely digital world, AR preserves and enhances the true environment.^3^ AR systems allow users to fuse digital and physical realities through superimposition of a software-generated image on the user’s view of the real world. They maintain orientation in a three-dimensional (3D) plane through a combination of global positioning systems, accelerometers, and gyroscopes.^2^ MR devices use sensors and spatial mapping technology to scan and interpret the true environment, allowing for a deeper integration of virtual and physical elements than AR commonly provides.

**FIG. 1.**
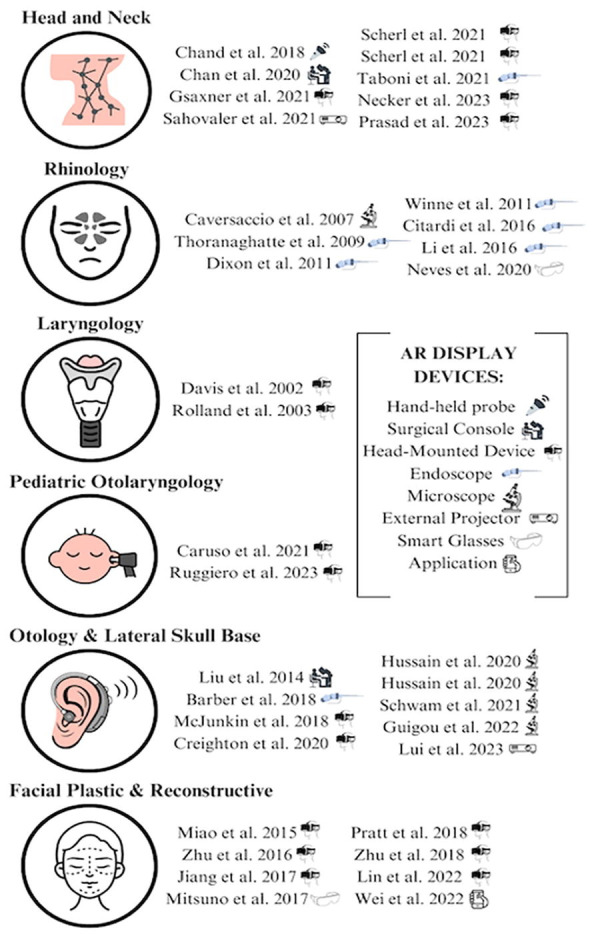
Summary of augmented reality display devices used in the studies reviewed.

Using these tools, users view a digital overlay in the form of virtual images, 3D models, textual annotations, labels, or animations displayed over the physical environment. As the user manipulates objects in the real world, the digital overlay adjusts accordingly, maintaining a synchronized view. Users can engage with AR through head-mounted devices (HMDs), smart glasses, handheld devices, endoscopes, microscopes, external projectors, and surgical consoles (Fig. 1).^4^ However, XR authorities may argue that the mixing of AR with other technologies such as external projectors and holographic overlays may not be considered true XR. In the following studies, most authors opted to use AR and certain elements of MR rather than VR because the end goal is to adopt these technologies in surgical practice. Users need to be able to maintain their view of the true environment, and thus, VR is not conducive.

Otolaryngological surgeries demand a thorough understanding of complex anatomical structures and their relationships to each other.^5,6^ Intraoperatively, surgeons carefully navigate complex anatomy, encountering bone, cartilage, muscle, nerves, blood vessels, mucosa, and glands, all of which compose the head and neck. Traditionally, surgical planning has been based on two-dimensional (2D) imaging such as computed tomography (CT), ultrasound (US), and magnetic resonance imaging (MRI) scans.^7^ In recent years, surgical guidance has improved because of technologies available to head and neck surgeons for intraoperative navigation (IN). In rhinology, endoscopic sinus surgery is a common treatment modality that is most commonly paired with image-guided navigation systems.^8^ Registration of a navigational tracking system to the endoscope allows for real-time determination of instrument position relative to surgical landmarks. In otologic surgery, microscopes are the primary modality for intraoperative visualization, although endoscopes have been increasingly used in recent years.^9^ Conventional image guidance systems have sparingly been used in otology owing to insufficient precision, with the proximity of critical nerves and blood vessels requiring sub millimetric accuracy.^9,10^ Endoscopes and microscopes have enabled surgeons to perform minimally invasive procedures, leading to reduced tissue damage and faster recovery times.^8–11^ However, a significant limitation of the 2D images produced by endoscopes and microscopes is the restricted sense of depth perception and the splitting of attention between the surgical field and the imaging screen.^11^

Head and neck cancer surgery is typically open, apart from oropharynx cancer surgery, which is often treated with minimally invasive transoral robotic surgery (TORS).^12^ Fluorescence guidance has also shown early value in head and neck cancer for the visualization of specific structures in tumor resection and sentinel lymph node mapping.^7^ Intraoperative nerve monitoring is widely used in thyroid and parathyroid surgery for neural mapping and in parotid surgery for facial nerve monitoring.^13^ These tools can aid in head and neck dissection and prognostication of postoperative neural function. Similarly, facial plastic surgery is often open with or without loupe magnification. Virtual surgical planning with 3D modeling is frequently used in head and neck reconstructive surgery to visualize and optimize oncologic, aesthetic, and functional goals.^14^

AR has the potential to improve these approaches by providing another layer of visualization for surgical planning, intraoperative guidance, and execution. Previous studies in surgical specialties such as plastic surgery,^15^ neurosurgery,^16,17^ and maxillofacial surgery^18,19^ have demonstrated the feasibility of AR applications and their role in improving operation time and precision. In this review, we explore the applications of AR in otolaryngology by specialty, highlighting the technologies used and their applications, limitations, and impact.

## Methods

### Criteria for inclusion

**FIG. 2.**
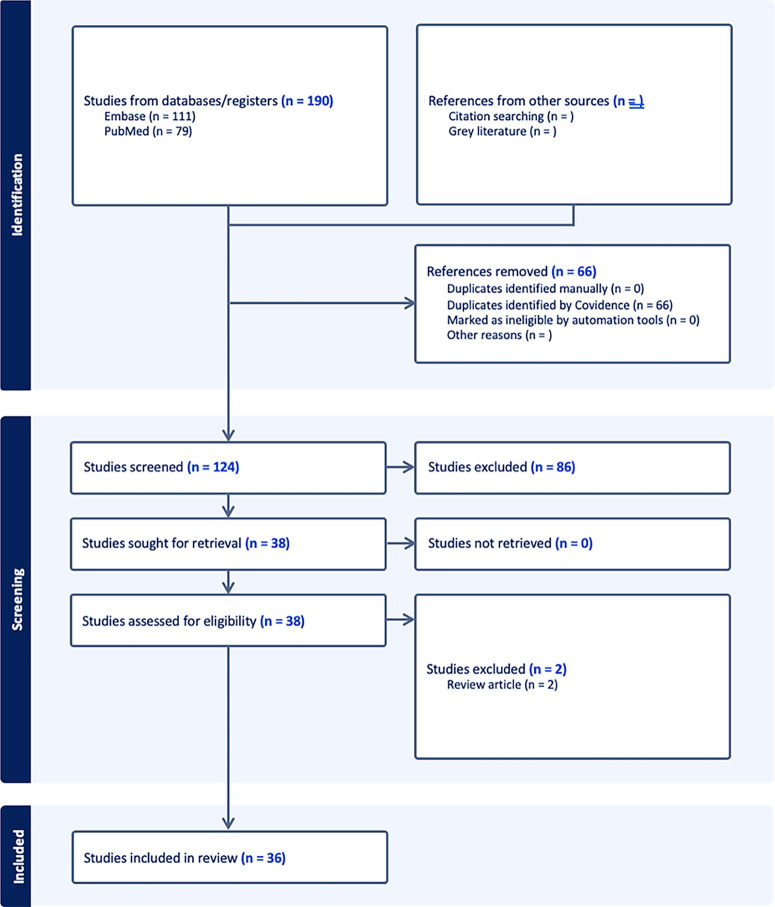
Augmented reality in otolaryngology scoping review flow diagram.

A literature review was conducted via PubMed and EMBASE according to the guidelines and recommendations of the Preferred Reporting Items for Systematic Reviews and Meta-Analyses extension for Scoping Reviews framework (Fig. 2). Given the novelty of the topic and variability in reporting of outcomes, a scoping review was determined to be suitable for the goal of this investigation. Articles were considered eligible for inclusion if they investigated the clinical application of an AR operating system or software in otolaryngology—head and neck surgery. Exclusion criteria included non-English articles, field of application other than otolaryngology, abstracts, and letters/commentaries.

### Search strategy

The following series of keywords were used as search terms: “augmented reality” OR “AR” AND “otolaryngology” OR “head and neck surgery” OR “ENT” OR “head and neck cancer” OR “rhinology” OR “laryngology” OR “pediatric otolaryngology” OR “otology” OR “skull base” OR “facial plastic surgery.” The primary authors (M.A. and F.A.) performed article collection and data entry. Predefined inclusion and exclusion criteria were used to select the eligible articles by September 2023. The review tool Covidence (Melbourne, Australia) was used to conduct the formal review. Articles were selected by author discretion based on relevance to the subtopics and adherence to inclusion criteria. Conflicts between reviewers were resolved through discussion.

## Results

### Search results

In total, 36 articles were included in this review. The initial search resulted in 190 results. Sixty-six duplicates were removed, and 86 articles were excluded after titles and abstracts were screened. Two full text review articles were excluded (Fig. 2). Findings were categorized and reported by subspecialty.

## Applications of AR in Otolaryngology Subspecialties

### Head and neck cancer

AR technologies have proven valuable in head and neck procedures, assisting in real-time tumor margin localization and guiding identification and navigation of critical anatomical structures during surgery. Chan et al. showcased an application of AR within TORS. Their cadaveric feasibility study used 3D virtual reconstructed models of the primary tumor derived from preoperative imaging to overlay the internal carotid artery.^20^ This study demonstrated the potential for AR to enhance critical structure identification during live surgical procedures. Comparably, Taboni et al. used 3D-rendered surgical navigation with virtual endoscopy on artificial skulls for transnasal delineation of maxillary tumors and their relationship to the carotid artery. In the unguided simulation, surgeons could only view cross-sectional images before starting, with no access to the real-time navigation. In the tumor-guided simulation, the virtual surgical tools were guided using real-time tool tracking and the 3D tumor and carotid imaging segmentation. Lastly, in the carotid-guided simulation, a 2-mm alert cloud surrounding the carotid was added to the tumor-guided setting to alert the user of proximity of the carotid. This approach signiﬁcantly improved the rate of margin-negative clearance around the tumor posteriorly (76% vs. 96% vs. 97.8%, unguided, tumor-guided, and carotid-guided cuts, *p* < 0.0001) and reduced damage to the carotid artery (6.7% vs. 0.9% vs. 1.0% unguided, tumor-guided, and carotid-guided cuts, *p* < 0.0001).^21^ In a preclinical study for open sinonasal tumor resections, Sahovaler et al. showed that among 335 simulated cuts in virtual osteotomies, using AR with IN significantly improved margin delineation. In unguided cuts, 20.7% had intratumoral cuts compared with 0% in AR + IN-guided cuts (*p* < 0.0001).^22^ Achieving clear surgical margins in head and neck cancer resections is imperative. These studies demonstrated uses of AR navigation that hold potential for improving margin status and, ultimately, head and neck cancer outcomes.

Building on prior cadaveric research, Scherl et al. demonstrated the feasibility of projecting holographic models via the Microsoft HoloLens 1^®^ (Microsoft Corporation, Redmond, WA, USA) onto patients undergoing live parotid surgery.^23,24^ Derived from MRI data, these 2D and 3D holographic models displayed a mean alignment error of 12.4 mm. Accuracy improved as more participants used the AR models, ultimately achieving a mean alignment error of 10.06 mm at the study’s conclusion. Although no specific parameters for accuracy in parotid surgery have been established, the mean alignment error is substantial and may be a limiting factor in the widespread adoption of this AR technology. Expanding the scope of AR surgical applications, Chand et al. described an innovative use of AR for sentinel node localization in oral squamous cell carcinoma.^25^ They used a 3D handheld AR scanning single-photon emission computed tomography (SPECT) probe intraoperatively to assess sentinel node alignment with preoperative SPECT-CT images. The SPECT probe’s localization matched preoperative imaging. Similarly, the fluorescence tracer localized to the same nodes identified in both SPECT-CT and handheld SPECT images, further validating the approach.

**FIG. 3.**
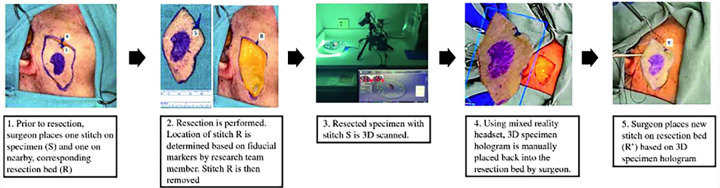
Accuracy study workflow. This figure is quoted with permission from Prasad et al.^26^

In a cadaveric study, Prasad et al. demonstrated the feasibility of using 3D specimen holograms to guide head and neck cancer reresections, reporting a mean relocation error of 4 mm.^26^ Using the Microsoft HoloLens 2^®^ (Microsoft Corporation), surgeons could manipulate, resize, and annotate 3D specimen holograms in real time, using hands-free gestures to maintain sterility in the operating room (OR) (Fig. 3). The average time for manual realignment of the 3D holographic specimen onto the cadaver was 4 min. Using analogous techniques, Necker et al. demonstrated the utility of overlaying annotated specimen holograms onto free flap harvest sites to aid in reconstructive planning. In this model, colored annotations corresponded with pathological markers and guided the orientation of the virtual 3D specimens onto the cadaver resection bed in an average time of 10 min.^27^ Similarly, Gsaxner et al. showed that physicians required an average training time of 12.7 ± 6.6 min to localize head and neck carcinoma in relation to the patient’s external anatomy using an AR HMD device.^28^

### Rhinology and anterior skull base

**FIG. 4.**
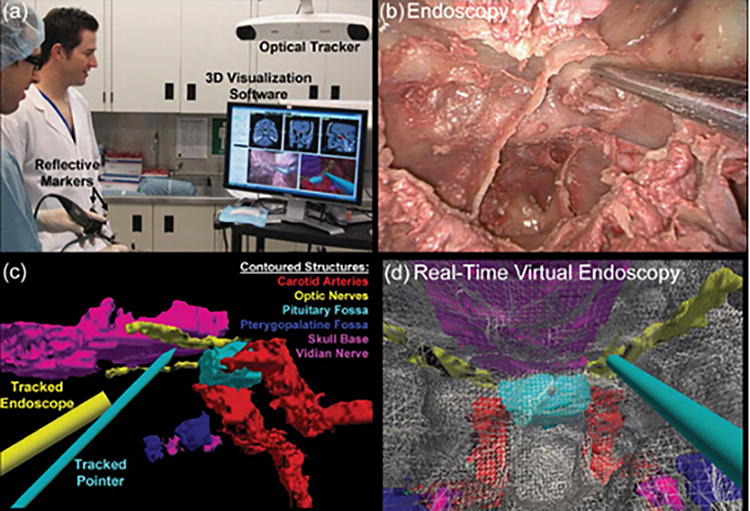
**(a)** Image-guided surgery system used for preclinical study, including optical tracking system Northern Digital Inc. (NDI; Polaris, Waterloo, Ontario, Canada), tracked endoscope and pointer, and custom three-dimensional visualization software. **(b)** Real endoscopic view of cadaveric anterior skull base region. **(c)** Surgical contours of critical anatomical structures in intraoperative cone-beam computer tomography (CT) image. **(d)** Real-time endoscope tracking and endoscopy-CT registration enables wall down perspective views of contoured structures aligned with real endoscopic view. This figure is quoted with permission from Dixon et al. (CC BY 4.0).^31^

**FIG. 5.**
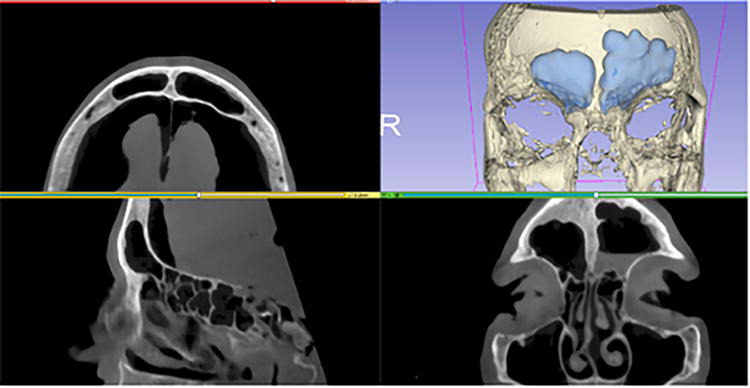
Preoperative CT scans and segmented structures (bone and frontal sinus) on 3D Splicer application. This figure is quoted with permission from Neves et al. (CC BY 4.0).^35^ 3D, 3 dimensional.

In rhinology, AR applications through endoscopic and microscopic displays enhanced sinus pathway navigation, showed anatomical boundaries, and reduced operation time and physician workload in various cadaveric and live surgeries. Li et al. investigated the feasibility of a novel AR-guided navigation system fusing endoscopic to 3D virtual images for endoscopic sinus and skull base surgery (Fig. 4).^29^ In this cadaveric study, they reported a target registration error of 1.28 ± 0.45 mm. This is compatible with existing parameters for image-guided endoscopic sinus surgery, which accept a 1.5–2 mm maximum registration error.^30^ The AR-guided navigation system led to reduced mental and physical demands, decreased time pressure, improved performance, reduced effort, and lower frustration levels compared to conventional navigation systems (*p* < 0.05), as shown by the National Aeronautics and Space Administration Task Load Index (NASA-TLX) rating scale. Average operation time was significantly decreased using the AR-guided navigation system (104.93 ± 24.61 vs. 88.27 ± 20.45 mins, *p* < 0.05). Similarly, Dixon et al. found that the AR-guided navigation system aided in endoscopic localization of skull base landmarks for 85% of participants and increased confidence in 97% (Fig. 5).^31^ In addition, they reported a significant reduction in mental effort, demand, and frustration based on the NASA-TLX (*p* < 0.05).

**FIG. 6.**
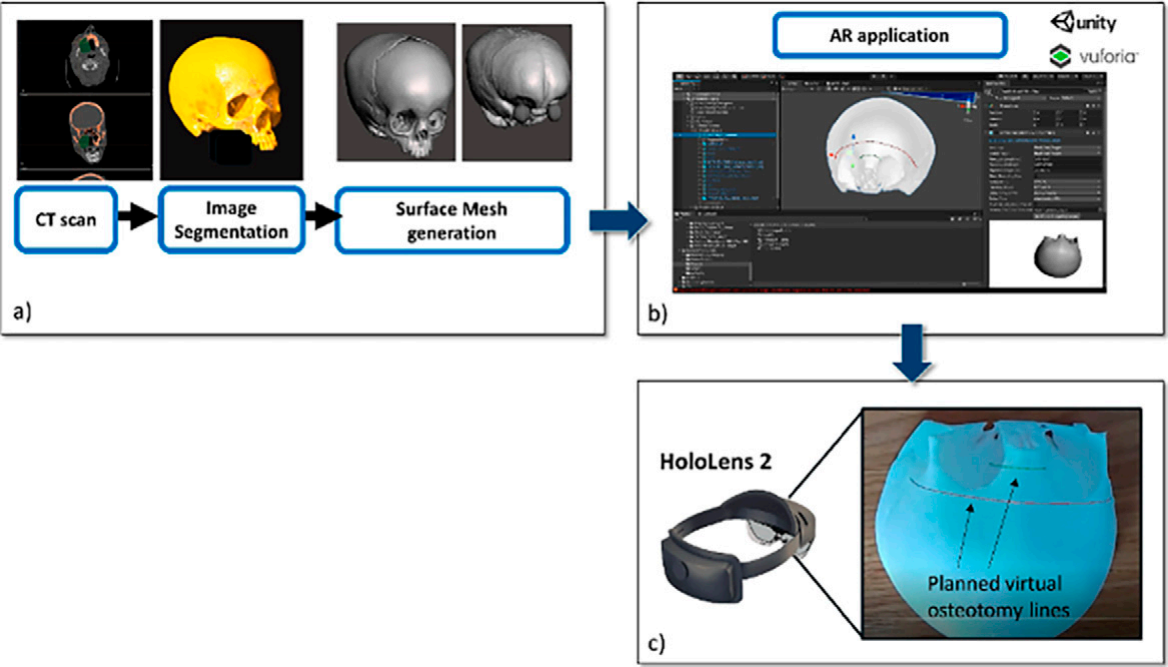
Development phase: **(a)** from CT scan to virtual content preparation; the virtual 3D skull model was also 3D printed to obtain a patient-specific phantom; **(b)** Unity software interface used for AR application development; **(c)** the planned osteotomy lines displayed in AR with HoloLens 2 smart glasses. This figure it quoted with permission from Ruggiero et al (CC BY 4.0).^37^ AR, augmented reality.

Citardi et al. created patient-specific virtual models of frontal recess structures and the frontal sinus outflow pathway for endoscopic sinus surgery.^32^ In cadaveric dissections, they used AR navigation to overlay the models onto the surgical field, effectively identifying the optic nerve and internal carotid artery before direct visualization in the surgical field. Surgical navigation achieved a target registration accuracy of 1.5 mm. Thoranaghette et al. conducted a cadaveric study investigating the use of AR to preprogram and project landmarks onto patients based on preoperative CT scans in endoscopic sinus surgeries.^33^ They reported a mean error of 2.25 mm. Comparably, Winne et al. highlighted the use of AR for cadaveric paranasal sinus surgical interventions, demonstrating an overlay error of 2.8 ± 1.0 mm.^34^ Notably, in both these studies, the target registration error is greater than the commonly accepted 2-mm maximum. Neves et al. constructed a frontal sinus hologram based on CT scan images to aid in the demarcation of frontal sinus boundaries for osteoplastic flaps (Fig. 6).^35^ The postprocedure CT scan images revealed a mean difference of 1.4 ± 4.1 mm between the contour of the osteotomy and the contour of the frontal sinus in cadaveric heads. A significant challenge of translating AR applications from cadaveric to human studies is the inherent difference in time restrictions and time under anesthesia for actual patient care. In cadaveric studies, there is more freedom to troubleshoot the technology than in live surgery. Although less prominent in sinus surgery, which focuses on osseous structures, cadaveric tissue cannot reflect physiological responses such as bleeding and tissue contraction, posing another difference between cadaveric and live surgery.

In a case series of 313 patients undergoing endoscopic anterior skull base surgical procedures, Caverasaccio et al. demonstrated that their AR-guided system was feasible in chronic sinusitis and nasal polyp interventions (*n* = 181), biopsies (*n* = 29), frontal sinus recess revision (*n* = 29), and tumor resections (*n* = 22).^36^ They reported a mean target error of <1 mm for direct overlay of structures onto the surgical microscopic view. Further investigation through randomized controlled clinical trials (RCTs) is necessary to definitively establish AR’s safety in rhinology.

### Pediatric otolaryngology

Limited research exists regarding AR use in pediatric otolaryngology, but it has proven valuable in enhancing patient comfort during live procedures and offering precise guidance for osteotomy cuts in cadaveric studies. In a preclinical study investigating the utility of AR in pediatric craniofacial surgery, Ruggiero et al. used the Microsoft HoloLens 2 to guide osteotomies on a patient-specific 3D-printed skull model.^37^ The model, based on preoperative CT scans, detailed bone, skin, orbits, and brain structures. Holograms were superimposed on the skull model to visualize structures and map trajectories for frontal and nasal osteotomies. With a success rate of 97%, users accurately traced the osteotomy trajectory within ±1.5 mm compared with standard cutting guides. This study pioneered a surgical application for AR in pediatric otolaryngology.

In a case series involving three patients aged 11–17 undergoing nasal endoscopy or transnasal laryngoscopy, Caruso et al. explored AR’s role in reducing patient fear and enhancing cooperation.^38^ Patients interacted with a game app on the Mira AR HMD (Mira Labs, Los Angeles, CA, USA) and virtually smashed fruits while the surgeon performed the procedure. All patients reported low fear (average: 0.33/4), high satisfaction (average: 4.2/5), and no pain (average: 0/10) when using AR during their procedures. Parents found AR to be a valuable distraction tool (average: 4.3/5), and all participants—patients, parents, and otolaryngologists—recommended its incorporation. As the development of AR applications progresses and their safety is demonstrated in adult populations, there will be an opportunity to expand the use of these applications to pediatric populations.

### Laryngology

To date, AR applications in laryngology have been limited to endotracheal intubation guidance. Davis et al. and Rolland et al. demonstrated similar AR applications in endotracheal intubation training. Using an HMD, virtual 3D internal airway anatomy was superimposed onto solid mannequins. They hypothesized that this AR application improved visualization and hand–eye coordination for endotracheal tube positioning.^39,40^ Published in 2002 and 2003, respectively, these studies delineated initial applications of AR in otolaryngology. Limited by availability and capability of AR technology at that time, they envisioned the future design of AR to include patient-specific 3D virtual models based on CT scans. To our knowledge, these methods have not yet been described in AR applications of laryngology. However, other otolaryngology subspecialties, such as rhinology^31,33,35^ and head and neck,^22,23,28^ have demonstrated the feasibility of AR guidance using patient-specific preoperative CT scans, highlighting a potential for use in laryngology.

### Otology and lateral skull base surgery

In otology and lateral skull base surgery, AR has predominantly been used for intraoperative landmark guidance and avoidance of critical structures. Lui et al. investigated the effect of AR-guided surgery on accuracy, duration, and ease of bone conduction device implantation.^41^ Pre- and postoperative CT scans were superimposed on cadaveric heads through AR projection. To assess the accuracy of the AR projection system, pre- and postoperative scans were superimposed to measure the distance between the planned site for cochlear device implantation and the actual site. This measurement was defined as “center-to-center” distance and was compared between the unguided and AR-guided arms of the study. Both operative time (4.3 ± 1.2 min vs. 6.6 ± 3.5 min, *p* = 0.030) and center-to-center distances (1.9 ± 1.6 mm vs. 9.0 ± 5.3 mm, *p* < 0.001) were significantly decreased in AR-guided implantation. There was an overall average error of 1.7 ± 0.6 mm between the bony fiducial markings and the AR projected fiducials. Liu et al. completed bilateral cadaveric mastoidectomies, posterior tympanostomies, and cochleostomies using the da Vinci Si system guided by AR.^42^ An osteon pneumatic drill was attached on the shaft of an 8-mm da Vinci Si tool and used to dissect the temporal bone. A 3D visualization was projected through the surgeon’s console using a 12-mm (0°) daVinci Si endoscope which was placed in parallel to the drill. Bilateral cochlear implants were successfully implanted with no violation of critical structures and with precise image overlay of the facial nerve, round window position, and basal turn of the cochlea. Postoperative cone beam CT scans confirmed successful placement of the implant electrode array into the basal turn of the cochlea.

**FIG. 7.**
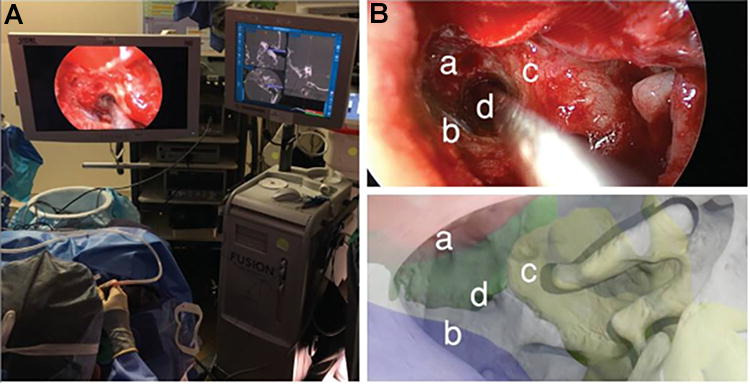
**(A)** Intraoperative photo of the live surgery performed using a transcanal endoscopic approach. **(B)** Comparison of intraoperative (upper panel) with virtual, preoperative otoendoscopic views (lower panel) demonstrated that the virtual render predicted the trajectory of the real surgical approach based on structures at risk: **(a)** internal carotid artery, **(b)** jugular bulb, **(c)** basal turn of the cochlea, and **(d)** access to petrous apex cyst. This figure is quoted with permission from Barber et al. (CC BY 4.0).^43^

Barber et al. used an AR navigation system in preoperative surgical planning for transcanal endoscopic drainage of a petrous apex cyst.^43^ The patient’s CT scan was manually segmented to isolate individual anatomical structures and create a 3D-printed model. Preoperatively, fiducial registration of the AR navigation system was done on the model with a 1:1 match to the original CT imaging and a 0.7 mm margin of error along landmarks, which is within the accepted parameter of <1 mm for image-guided otologic surgery.^44^ The virtual otoendoscopic view predicted the trajectory of the carotid artery, jugular bulb, and cochlea on the model and was found to be accurate intraoperatively (Fig. 7). Comparably, Hussain et al. established the feasibility of an intraoperative AR system that merged real-time microscope video with a 2D CT-based virtual image for visualization of middle ear cleft structures during transtympanic procedures in nine patients.^45^ The overlay demonstrated minimal fiducial and target registration errors, measuring 0.38 ± 0.23 mm and 0.36 ± 0.15 mm, respectively. Using analogous methods, Hussain et al. and Guigou et al. developed additional AR applications for intraoperative guidance. One study evaluated an AR system for visualization of the cochlear axis during transmodiolar auditory nerve implantation and reported a 0.31 ± 0.10 mm and 15.10 ± 1.28 mm target registration error for the cochlear apex and axis,^9^ respectively. The other used AR to guide middle ear surgery through a marker-based system with mean localization errors of 0.56 ± 0.14 mm for the umbo, 0.54 ± 0.16 mm for the incus tip, and 0.46 ± 0.19 mm for the round window niche.^46^

An AR-based HMD was used by Creighton et al. to manipulate temporal bone phantom models, displaying CT renderings for guiding real-time rotation alignment.^47^ Despite successful display and manipulation, they concluded that the target registration error of 10.62 ± 5.90 mm was too large for safe use in lateral skull base surgery. McJunkin et al. applied comparable techniques to create interactive 3D holograms encompassing soft tissue, bony anatomy, and internal ear structures within cadaveric temporal bone models and yielded an average target registration error of 5.76 mm ± 0.54.^5^

**FIG. 8.**
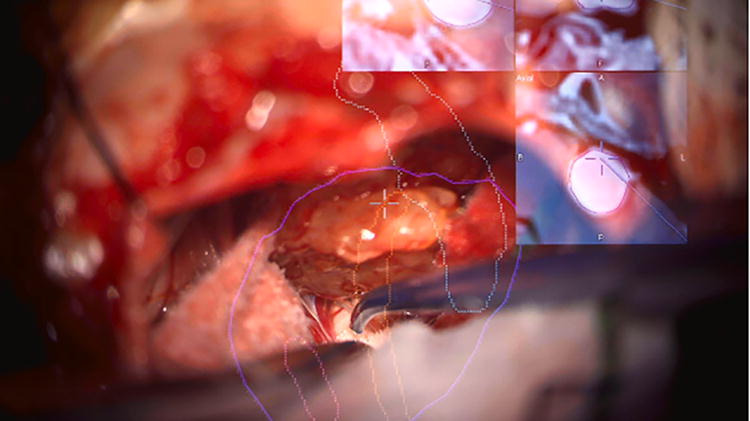
A screenshot of the heads-up-display from left vestibular schwannoma patient. The MRI images are projected into the top right corner of the screen and can be added or removed as needed. Segmented structures are highlighted above, namely, the tumor (purple) and a nerve believed to be the facial nerve (orange). This figure is quoted with permission from Scwham et al.^10^ MRI, magnetic resonance imaging.

AR guidance has also been used intraoperatively for cerebellopontine angle tumor resections (*n* = 40).^10^ Schwam et el. anecdotally reported AR projection of critical structures to be the most helpful in preparing the angle of approach, optimizing the skin incision, and maximizing the craniotomy through visualization of the dural venous sinuses. Although objective measures of AR-guided accuracy were not reported, this study demonstrated the feasibility of incorporating live AR guidance into lateral skull base surgery (Fig. 8). In otology and lateral skull base surgery, a substantial body of evidence demonstrates AR’s efficacy in cadaveric studies. However, additional prospective exploration in live surgical settings is essential to evaluate AR’s accuracy and its potential to enhance surgical preparation and outcomes.

### Facial plastics and reconstructive surgery

The use of AR in facial plastic and reconstructive surgery has been demonstrated in all stages of care, including preoperative planning, intraoperative guidance, and postoperative assessment of outcomes. In reconstructive surgery, AR has been used intraoperatively to identify surgical landmarks such as vascular pedicles, crucial to reconstructing Mohs defects. In exploration of feasibility, Jiang et al. designed an AR-based guidance technique that uses preoperative computed tomography angiography (CTA) imaging to overlay a hologram of the patient’s vasculature before making the incision.^48^ Similarly, Pratt et al. compared AR with conventional doppler US to find vascular pedicles for flap reconstruction.^49^ Qualitative feedback from the surgical team suggested that AR may be more reliable and less time intensive than traditional methods. Given that soft tissue structures are mobile and can more easily change position during a procedure, there has been more success in adapting AR to procedures involving fixed bony landmarks.

In 12 patients with orbital hypertelorism, Zhu et al. used a CT-based AR system to overlay preoperatively planned osteotomies to guide positioning of the orbits.^50^ Using this AR guidance, the authors reported no significant differences between the preoperative plan and the postoperative osteotomy outcome with a discrepancy of less than 2.5 mm. Unlike other subspecialties, the acceptable parameters for registration error in facial plastic surgery have not been well defined. However, the authors found this discrepancy to be negligible in their study. In addition, in a RCT by Qu et al., 20 patients with hemifacial microsomia undergoing mandibular distraction osteogenesis were divided into two groups, the conventional procedure and AR assistance to bring the preoperative planned planes for osteotomy directly into the surgical field and to assist with intraoral distractor placement.^51^ AR navigation significantly improved the accuracy of the osteotomy compared with the conventional approach; a limitation of the experiment was a lack of surgeon usability. However, with adequate registration, the surgeons were not only able to achieve an actual osteotomy closer to the preoperative plan, but they were also able to visualize the osteotomy line in real time.

**FIG. 9.**
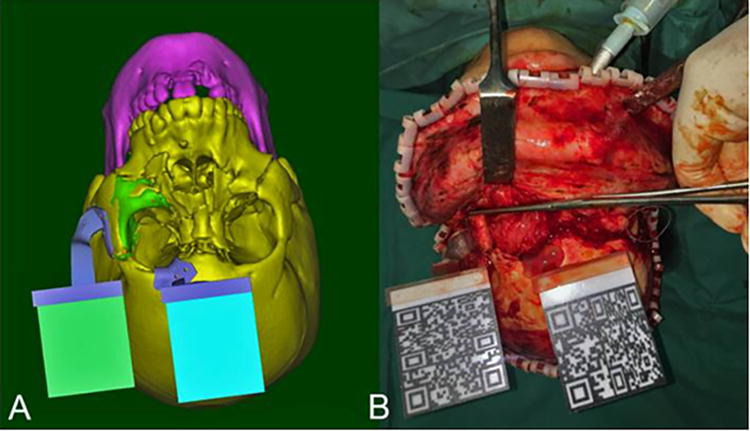
Preoperational design and the matched augmented reality in surgery by 3D printing plates. (**A)** Preoperational surgical design. (**B)** Three-dimensional printing tracking plate in the operation for real-time augmented reality tracking system. This figure is quoted with permission from Lin et al. (CC BY 4.0).^53^

Furthermore, Zhu et al. in a retrospective study of 93 patients with mandibular angle hypertrophy compared osteotomy with an AR-guided navigation system with 3D-printed individualized templates (IT) to a free-hand approach.^52^ Patients in the free hand group had lower preoperative planning time (*p* < 0.05) but no significant difference in operating room time (*p* > 0.05). Furthermore, the AR and IT groups showed reduced discrepancy between the planned and the actual osteotomy line (*p* < 0.01). Overall, this study demonstrated that AR-guided techniques could be reliable, accurate, and user friendly, without increasing time spent in the OR for complex craniofacial procedures. In addition, in an ongoing RCT, Lin et al. are comparing traditional optical navigation with AR navigation for the surgical treatment of complex zygomaticomaxillary fracture (Fig. 9).^53^ Eleven patients will be enrolled in each arm of the study. This study will evaluate the accuracy of the navigation systems, comparing between preoperative surgical plan and postoperative outcome. The shifting paradigm toward RCTs will help establish the validity of AR applications, creating opportunity for more widespread use.

AR has also been utilized in aesthetic surgery. Mitsuno et al. explored the utility of AR in body surface contouring via overlaying both preoperative (start) and “ideal postoperative” (goal) 3D images over patients intraoperatively.^54^ In a series of procedures, the authors evaluated the utility of 3D overlayed images to use as references to evaluate postoperative outcomes after zygomatic reduction, rhinoplasty for cleft nasal deformity, and ostectomy in patients with fibrous dysplasia. During the case, the authors were able to modify the transparency and color of all reference images to suit the individual surgeon’s desire and need. In all cases within this study, the actual postoperative outcome matched the “ideal postoperative” goal hologram with direct visual comparison. Outside of the operating room, Wei et al. developed an AR app that could assess for symmetry using facial landmarks powered by Google Face API.^55^ The application tracked and compared 10 facial landmarks and used those to assess for facial symmetry. To increase user friendliness, their AR app also used interactive measurement tools to enhance the user’s ability to assess for symmetry.

In summary, these studies demonstrate the multiple applications of AR in facial plastic and reconstructive surgery. Opportunities to innovate procedures such as rhinoplasty, facial trauma, and other facial plastics procedures where symmetry is a metric used in assessing success make AR an area for potential investigation and further studies. Programs already exist to assess various aspects of cosmesis and symmetry, and AR may allow physicians to effectively bring those tools into the operating room and onto the surgical field in real time.

## Discussion

### Limitations

Although AR tools have demonstrated great utility in otolaryngology, there are many limitations that must be considered. The individualized nature of each of these studies and their AR systems limits the validity and potential clinical use. Similarly, most studies were single-institution investigations with a small sample size and a novel AR system. Although cadaveric studies provide valuable insights, the transition from controlled experiments to the complexities of live surgeries presents a significant challenge. The unpredictability of surgical procedures, along with unforeseen complications, highlights the need for further exploration and adaptation of AR technology in live surgical settings. Furthermore, the accuracy of AR visualization heavily relies on the precision of tracking technology. Inaccurate tracking can result in misalignment between virtual and real-world objects, potentially compromising surgical guidance and decision-making. In the studies reviewed, the smallest target registration error was 0.36 mm in microscopic overlays of inner ear structures^45^ and reached 10.62 mm in an HMD display in lateral skull base surgery.^47^ The use of AR in surgery necessitates validation of its effectiveness and safety through rigorous clinical trials. Regulatory approval and standardization processes can be time-consuming and rigorous. Although AR tools supplement surgical preparation and execution, they remain secondary to a surgeon’s rigorous training and comprehensive understanding of anatomy.

Despite the proposed benefit of a unified surgical view through a single AR display, there have not been adequate studies evaluating whether this alters or improves surgical guidance and outcomes compared to the standard of care. It is important to acknowledge that a careful balance of visual information should be displayed, limiting the complexity of AR images that can lead to visual clutter, potentially causing distractions and confusion. In addition, the authors hypothesize that until the bulkiness of HMDs improves, AR will not be widely used in the operating room. Another limitation for AR technology in surgical practice is the learning curve. Surgeons and medical personnel must familiarize themselves with AR interfaces, controls, and procedures, requiring training to navigate the technology effectively and integrate augmented information into their workflow. Balancing existing surgical skills with new AR competencies is essential, and it is important to acknowledge that the learning curve may temporarily disrupt the established workflow. Many studies have demonstrated the feasibility of the integration of AR, but more investigation is required to determine whether the benefits of enhanced precision and decision-making outweigh initial challenges. Ultimately, this underscores the importance of maintaining patient safety as the highest priority.

Cost is another major consideration for the incorporation of AR. The cost of commercial AR systems can vary significantly based on the complexity of the system, the intended applications, the level of hardware and software integration, and the brand. Basic AR devices such as smart glasses or entry-level headsets range from $200 to $500, whereas advanced AR systems featuring robust tracking, complex software integration, and enhanced features can cost thousands per unit. This dynamic can potentially exacerbate health care inequalities, restricting access to financially capable health care settings.

## Conclusion

The integration of AR into otolaryngology offers a wide range of promising applications in preoperative planning, IN, and education. Across multiple subspecialties, numerous studies have demonstrated the feasibility of various AR programs, meeting the standards of acceptable accuracy levels. The future directions for integrating AR into otolaryngology surgical practice are promising and multifaceted. AR-enabled surgical platforms have the potential for remote collaboration, which would allow experts to provide guidance to surgeons in real time despite geographical distance and barriers. This potential application for AR-based collaboration could enable experts to contribute to surgeries in underserved regions or collaborate across international medical communities. In addition, AR has shown tremendous potential for use in otolaryngology surgical training. Using VR, MR, or AR tools to create realistic simulation environments to practice procedures and enhance surgical skills, surgical trainees could receive step-by-step guidance during simulations with overlay instructions, anatomical labels, or interactive guides directly projected onto their field of view. This function of AR tools could widen the range of surgical scenarios that trainees are exposed to, including rare or complex cases that may not otherwise be available during training. The current and potential uses of AR in otolaryngology are both exciting and diverse, showing great promise for enhancing otolaryngology head and neck practice.

## Abbreviation Used

3D: 3-dimensional
3D-SNVE: 3D surgical navigation with virtual endoscopy
AR: Augmented reality
CT: Computed tomography
CTA: Computed tomography angiography
HMD: Head-mounted device
IT: Individualized template
MR: Mixed reality
MRI: Magnetic resonance imaging
NASA-TLX: National aeronautics and space administration task load index
OR: Operating room
RCT: Randomized control trial
SPECT: Single photon emission computed tomography
TORS: Transoral robotic surgery
US: Ultrasound
VR: Virtual reality
XR: Extended reality
